# Chinese version of the Tendency to Avoid Physical Activity and Sport (TAPAS) scale: testing unidimensionality, measurement invariance, concurrent validity, and known-group validity among Taiwanese youths

**DOI:** 10.1186/s40359-024-01870-y

**Published:** 2024-07-08

**Authors:** Yi-Ching Lin, Jung-Sheng Chen, Nadia Bevan, Kerry S. O’Brien, Carol Strong, Meng-Che Tsai, Xavier C. C. Fung, Ji-Kang Chen, I-Ching Lin, Janet D. Latner, Chung-Ying Lin

**Affiliations:** 1 Department of Early Childhood and Family Education, National Taipei University of Education, Taipei, Taiwan; 2 Department of Medical Research, E-Da Hospital, I-Shou University, Kaohsiung, Taiwan; 3 School of Social Sciences, Monash University, Melbourne, 3800 Australia; 4 Department of Public Health, College of Medicine, National Cheng Kung University, Tainan, Taiwan; 5 Department of Pediatrics, College of Medicine, National Cheng Kung University Hospital, National Cheng Kung University, Tainan, Taiwan; 6 Department of Medical Humanities and Social Medicine, College of Medicine, National Cheng Kung University, Tainan, Taiwan; 7 Department of Rehabilitation Sciences, Faculty of Health and Social Sciences, The Hong Kong Polytechnic University, Hung Hom, Hong Kong China; 8 Department of Social Work, Chinese University of Hong Kong, New Territories, Hong Kong China; 9 Department of Healthcare Administration, Department of Family Medicine, Asia University, Taichung, 41354 Taiwan; 10 Department of Family Medicine, Asia University Hospital, Taichung, 41354 Taiwan; 11 Department of Kinesiology, Health, and Leisure, Chienkuo Technology University, Changhua, 50094 Taiwan; 12 Department of Psychology, University of Hawaii, Honolulu, HI 96822 USA; 13 Institute of Allied Health Sciences, Departments of Occupational Therapy and Public Health, and Biostatistics Consulting Center, College of Medicine, National Cheng Kung University Hospital, National Cheng Kung University, 1 University Rd, Tainan, 701401 Taiwan; 14 University of Religions and Denominations, Qom, Iran; 15 Biostatistics Consulting Center, College of Medicine, National Cheng Kung University Hospital, National Cheng Kung University, Tainan, Taiwan; 16 Faculty of Nursing, Universitas Padjadjaran, Sumedang, Indonesia

**Keywords:** Factor analysis, Physical activity, Reliability, Taiwan, Young adults

## Abstract

**Background and objectives:**

Psychosocial factors affect individuals’ desire for physical activity. A newly developed instrument (Tendency to Avoid Physical Activity and Sport; TAPAS) has been designed to assess the avoidance of physical activity. Considering cultural differences could be decisive factors, the present study aimed to translate and validate the TAPAS into Chinese (Mandarin) for Taiwanese youths, and further cultural comparisons are expected.

**Methods:**

Standard translation procedure (i.e., forward translation, back translation, and reconciliation) was used to translate the English TAPAS into the Chinese TAPAS. Following translation, 608 youths (mean [SD] age 29.10 [6.36] years; 333 [54.8%] women) participated in the study via a snowballing sampling method with an online survey. All participants completed the Chinese TAPAS and additional instruments assessing weight stigma and psychological distress. Confirmatory factor analysis (CFA) was used to examine the factor structure of the Chinese TAPAS and multigroup CFA to examine measurement invariance across gender (men vs. women) and weight status (overweight vs. non-overweight). Pearson correlations were used to examine the concurrent validity; independent t-tests between gender groups and weight status groups were used to examine the known-group validity.

**Results:**

Consistent with the English version, the Chinese TAPAS was found to have a one-factor structure evidenced by CFA results. The structure was invariant across gender and weight status groups evidenced by multigroup CFA results. Concurrent validity was supported by significant associations with the related constructs assessed (*r* = 0.326 to 0.676; *p* < 0.001). Known-group validity was supported by the significant differences in TAPAS total scores between gender and weight status groups (*p* = 0.004 and < 0.001; Cohen’s *d* = 0.24 and 0.48).

**Conclusion:**

The Chinese version of the TAPAS is a valid and reliable instrument assessing individuals’ avoidance of physical activity and sports due to underlying psychosocial issues among Taiwanese youths. It is anticipated to be applied within a large Asian population, as well as cross-cultural comparisons, for further explorations in health, behavioral and epidemiological research and practice.

## Introduction

The benefits of physical activity engagement have been well documented: physical activity is one of the most cost-effective methods to improve general health and prevent physical health conditions [1, 2] as well as chronic diseases, such as overweight (or obesity) and cardiovascular disease [3, 4]. Further, physical activity enhances cognitive and mental health and decreases the risk of mental conditions such as depression, anxiety, and panic disorders [5, 6]. Therefore, healthcare providers and governments need to focus on improving the physical activity levels of the general population.

Although the benefits of physical activity are well acknowledged worldwide by healthcare providers and governments, barriers to physical activity engagement have been well documented [7–9]. For example, a lack of accessibility for physical activity may decrease individuals’ capability to engage in exercise, or a lack of time may prevent individuals from participating in sports or exercise [7, 10]. Some barriers can be easily tackled by healthcare providers and related stakeholders (e.g., improving public facilities for physical activity engagement and helping people to improve time management for securing time for physical activity engagement). However, some psychosocial barriers may be harder to address, such as motivation, reluctance or avoiding physical activities.

Individuals’ reluctance and avoidance of physical activity are suggested as key factors to greater participation [11]. As such, a better understanding of the underlying cognitions for physical activity avoidance could help healthcare providers design more effective programs to enhance physical activity engagement [12] and address the actual factors causing the tendency to avoid physical activity and sports [11, 13], for example, weight or body image issues. Weight stigma and concerns about physical appearance are important factors associated with individuals’ physical activity engagement [12, 14–16]. Weight stigma is defined as weight-related discriminatory behaviors and ideologies towards people who have concerns about their weight and size, including weight-related self-stigma and perceived weight stigma [17]. Weight-stigmatized individuals frequently experience negative perceptions, stereotypes, and exclusion. An overweight body is often misjudged as reflecting personal health and physical activity inadequacies, potentially leading to decreased participation and motivation in physical activities [18, 19]. Given these factors, the prevalence of weight stigma, notably impacting mental and physical health, emphasizes the need to investigate its occurrence, especially in sports and exercise environments known for weight discrimination [20, 21]. In addition, body dissatisfaction includes negative cognitions and emotions toward individuals’ body images [22]. Body dissatisfaction has been positively associated with eating disorders, emotional disorders, suicidal attempts, impaired well-being, and physical activity avoidance [23–25]. The correlation between body dissatisfaction and adverse health outcomes may be attributed to feelings of shame and experiences of social isolation [26]. Previous studies suggest that negative self-perceptions, such as body dissatisfaction, commonly originate in environments that emphasize physical evaluation and physique-centric physical pursuits. There is a noted escalation of shame associated with body dissatisfaction, which has been linked with decreased engagement in physical activity and less-than-optimal athletic experiences [27]. However, there has been very little evidence on how weight stigma and body image issues may impact the tendency to avoid physical activity. To address the research gap, a new scale named the Tendency to Avoid Physical Activity and Sport (TAPAS) scale has been developed [11, 12] to assess avoidance of physical activity due to psychosocial factors such as weight stigma and body image issues.

The Consensus-based Standards for selecting health status Measurement Instruments (COSMIN) guide [28] was applied to construct the TAPAS as a psychometric instrument. Weight stigma and body image experts followed the traditional scale development guidelines [29–31] to generate items assessing this concept. After a rigorous mixed-method approach, the initial pool of TAPAS included 21 items for psychometric testing, and the finalized TAPAS included 10 items with satisfactory item properties (e.g., high factor loadings). Moreover, the 10-item TAPAS was found to be a one-factor structure with satisfactory concurrent validity (with weight stigma; *r* = 0.49) and known-group validity (women had significantly higher TAPAS score than men; Cohen’s d = 0.49) on an English-speaking (Australian) population [12]. However, the TAPAS has never been examined for its psychometric properties in Taiwanese ethnic populations. The barriers to engaging in physical activity and sports might vary considerably depending on cultural factors [32, 33]. For example, people from the West engage in physical activities mainly for competition and skill improvement, while people from the East consider social affiliation and wellness as the main reasons for participating in sports. Therefore, it is essential to establish whether the TAPAS retains good psychometric properties in other ethnic populations, such as Asian people and languages (i.e., Taiwanese in the present study).

The purpose of the present study is to translate the English version of the TAPAS into a Traditional Chinese version and to examine the psychometric properties of the Chinese TAPAS among Taiwanese youths. The following psychometric properties were examined: (i) the factor structure of the TAPAS and its hypothesized one-factor structure; (ii) internal consistency/reliability of the TAPAS; (iii) measurement invariance of the TAPAS, with an assumption that the TAPAS is invariant across gender and weight status; (iv) concurrent validity of the TAPAS, with a hypothesized relationships between the TAPAS and associated constructs related to weight-related self-stigma, perceived weight stigma, and psychological distress; and (v) known-group validity of the TAPAS, and we hypothesized that the TAPAS could effectively distinguish the differences of avoidance of physical activity and sport between genders (i.e., men vs. women) and between weight status groups (i.e., overweight vs. non-overweight). Once a reliable and validated Chinese version of TAPAS is developed, it is anticipated to be applied within a large Asian population and cross-cultural comparisons for further understanding in health behavioral and epidemiological research and practice.

## Methods

### Participants and recruitment procedure

The National Cheng Kung University Human Research Ethics Committee (Approval No. NCKU HREC-E-110-486-2) and the National Cheng Kung University Hospital Institute of Review Board (IRB No. A-ER-111-445) approved the study protocol. Data was collected using snowballing sampling via the online survey platform of *Survey Monkey*, and at least 400 participants (200 overweight and 200 non-overweight) were required for the present study [34]. Firstly, the online survey link was distributed with the assistance of faculty members in several universities in Taiwan (including universities located in Northern [1 university], Central [1 university], and Southern Taiwan [2 universities]).

The participants who received the survey link were encouraged to share it with their friends or colleagues. The inclusion criteria of the target participants were (i) aged between 20 and 40 years; (ii) able to read traditional Chinese characters; (iii) having access to complete the online survey; and (iv) providing the e-form consent for participation. Data were collected between 31 August and 31 December 2022. *Survey Monkey* identified the participants’ IPs to avoid the same participant completing the online survey more than once. In addition, an experienced research assistant cleaned the data via the following methods: (i) if the same email addresses and phone numbers were used more than once; (ii) if there were unreasonable values (e.g., over 200 cm for height).

### Translation of the Tendency to Avoid Physical Activity and Sport (TAPAS) scale

We used a standard translation procedure to translate the English TAPAS into the Chinese TAPAS with traditional Chinese characters [35]. In the first step, two bilingual translators who were native Chinese speakers with expertise in English independently conducted forward translation (i.e., from English to Chinese). In the second step, the two forward translators worked with the corresponding author to review the two forward-translated versions of TAPAS and generated a reconciled Chinese version of TAPAS. In the third step, a bilingual translator who was a native Chinese speaker with expertise in English and was unaware of the original TAPAS back-translated the reconciled Chinese version of TAPAS into English. In the fourth step, the two forward translators, the back translator, and the corresponding author had a panel meeting with other researchers with expertise in pediatrics, public health, psychometrics, physical activity, and weight to generate a final draft of the Chinese version of TAPAS. In the last step, three university students were invited to read the final draft (pre-survey) of the Chinese version of TAPAS for wording suggestions to finalize the Chinese version of TAPAS for formal psychometric evaluation. The students were given instructions on how to evaluate the wordings in Taiwanese culture. All three students agreed that the final draft of the Chinese version of TAPAS was readable without difficulties. Since the students suggested that no items required changes, no changes were made to the final draft. Moreover, the original developers (who are also the present study’s coauthors) assessed the Chinese version of TAPAS to ensure its equivalence to the original TAPAS in four levels: semantic, idiomatic, experiential, and conceptual.

### Measures for concurrent validity (please see appendix for all the measures’ details)

#### Demographic measures

The participants were asked for the following demographic information: age (reported in years), gender (reported in man, woman, or others), height (reported in cm), and weight (reported in kg). One item asks how many days a week the participants engaged in physical activity where breathing is somewhat harder than normal.

### Tendency to Avoid Physical Activity and Sport (TAPAS) scale

The TAPAS is a 10-item instrument assessing the tendency levels of an individual avoiding participating in physical activity and sport due to weight or appearance concerns. Each TAPAS item was rated using a five-point Likert scale (1 = strongly disagree; 5 = strongly agree) and a total TAPAS score summed the 10 TAPAS item scores (total score between 10 and 50). A higher TAPAS total score indicates a higher level of tendency to avoid physical activity and sport.^11^ The original TAPAS is an English version with good internal consistency (Cronbach’s α = 0.94) [12].

### Weight Bias Internalization Scale (WBIS)

The WBIS is an 11-item instrument assessing the level of weight-related self-stigma. Each WBIS item was rated using a five-point Likert scale (1 = strongly disagree; 5 = strongly agree), and a total WBIS score summed the 11 WBIS item scores (total score between 11 and 55). A higher WBIS total score indicates a higher level of weight-related self-stigma [36, 37]. The original WBIS is an English version with good internal consistency (Cronbach’s α = 0.90) [36]. The present study used the Chinese version of WBIS, which has good psychometric properties [38, 39]. Moreover, the internal consistency of the WBIS used in the present sample was satisfactory: Cronbach’s α = 0.82; McDonald’s ω = 0.87.

### Weight Self-Stigma Questionnaire (WSSQ)

The WSSQ is a 12-item instrument that also assesses the level of weight-related self-stigma. Each WSSQ item was rated using a five-point Likert scale (1 = strongly disagree; 5 = strongly agree), and a total WSSQ score summed the 12 WSSQ item scores (total score between 12 and 60). A higher WSSQ total score indicates a higher level of weight-related self-stigma [40, 41]. The original WSSQ is an English version with good internal consistency (Cronbach’s α = 0.88) [40]. The present study used the Chinese version of WSSQ, which has good psychometric properties [39, 42]. Moreover, the internal consistency of the WSSQ used in the present sample was satisfactory: Cronbach’s α = 0.92; McDonald’s ω = 0.92.

#### Perceived Weight Stigma Scale (PWSS)

The PWSS is a 10-item instrument assessing the level of perceived weight stigma. Each PWSS item was rated using a dichotomized scale (0 = no; 1 = yes), and a total PWSS score summed the 10 PWSS item scores (total score between 0 and 10). A higher PWSS total score indicates a higher level of perceived weight stigma [43–45]. The PWSS has been validated in different language versions with good internal consistency (e.g., Cronbach’s α = 0.85 in the Thai version; 0.90 in the Indonesian version) [17, 46–48]. The present study used the Chinese version of PWSS, which has good psychometric properties [49–51]. Moreover, the internal consistency of the PWSS used in the present sample was satisfactory: Cronbach’s α = 0.86; McDonald’s ω = 0.87.

### 21-item Depression, Anxiety, Stress Scale (DASS-21)

The DASS-21 is a 21-item instrument assessing the level of psychological distress. Each DASS-21 item was rated using a four-point Likert scale (0 = did not apply to me at all; 3 = applied to me very much or most of the time) and a total DASS-21 score summed the 21 DASS-21 item scores (total score between 0 and 63). A higher DASS-21 total score indicates a higher level of psychological distress [52]. The original DASS-21 is an English version with good internal consistency (Cronbach’s α = 0.80 to 0.91) [53]. The present study used the Chinese version of DASS-21, which has good psychometric properties [54]. Moreover, the internal consistency of the DASS-21 used in the present sample was satisfactory: Cronbach’s α = 0.94; McDonald’s ω = 0.94.

### Statistical analysis

For the participants’ weight status, their weight and height information was used to calculate body mass index (BMI; kg/m^2^) and classify the participants into overweight (> 24 kg/m^2^) or non-overweight according to Taiwanese norms [55]. For the psychometric testing, internal consistency, factor structure (i.e., if the TAPAS is a one-factor structure), measurement invariance, concurrent validity, and known-group validity of the TAPAS were examined. In addition to the psychometric testing, a mediation model was performed to examine the mediator role of TAPAS in the association between weight status and psychological distress. The internal consistency was conducted using the *psych* package in the R software, factor structure and measurement invariance using the lavaan package in the R software, concurrent validity, and known-group validity using the IBM SPSS version 20.0. The mediation model was performed using the Hayes’ Process Macro (Model 4) under the SPSS 20.0 version.

Internal consistency was assessed using Cronbach’s α and McDonald’s ω; both α and ω values larger than 0.7 indicate good internal consistency [56]. The one-factor structure of the TAPAS was examined using confirmatory factor analysis (CFA) with a diagonally weighted least squares (DWLS) estimator. The DWLS estimator was used because it is an estimator suitable for categorical scales, such as the Likert scale used in the TAPAS. We directly used CFA to test a unidimensional structure because the original TAPAS was found to have a one-factor structure from the results of an exploratory factor analysis (EFA) [12]. Although some may argue that a translated instrument needs to be tested using EFA, we considered that using CFA is better because the TAPAS also has a theoretical background and was verified by experts in this field to be a unidimensional structure. Considering the unidimensional evidence of the English TAPAS, we consider that directly testing CFA is appropriate. In the CFA, the following fit indices were used to examine if the present data fit the one-factor structure of the TAPAS: comparative fit index (CFI) and Tucker-Lewis index (TLI) > 0.9; root mean square error of approximation (RMSEA) and standardized root mean square residual (SRMR) < 0.08; and a non-significant χ^2^ test [17, 57, 58].

Multigroup CFA was then used to examine if the one-factor structure of the TAPAS is invariant across gender (i.e., men vs. women) and across weight status (i.e., overweight vs. non-overweight). Three nested models were constructed for the invariance test [59]: Configural model (all the factor loadings and item intercepts were not constrained across groups), metric invariance model (only factor loadings but not item intercepts were constrained equal across groups), and scalar invariance model (both factor loadings and item intercepts were constrained equal across groups). Fit indices of ΔCFI, ΔTLI, ΔRMSEA, and ΔSRMR were then used to examine if the invariance is supported. When ΔCFI and ΔTLI > -0.01 together with ΔRMSEA and ΔSRMR < 0.01, the invariance is supported [47, 60, 61]. However, ΔRMSEA < 0.03 is proposed to be acceptable to claim metric invariance.

The present study aimed to recruit more than 200 participants who were overweight and more than 200 who were not overweight because the CFA required a sample size of over 200 to make an unbiased estimation [34]. Because the present study tested measurement invariance across weight status using the multigroup CFA, both subgroups (i.e., overweight subgroup and non-overweight subgroup) were needed to satisfy the minimal requirements for CFA.

Because the original TAPAS was found to be associated with weight stigma [12], we hypothesized that the Chinese version of TAPAS would be associated with weight stigma measures (including WBIS, WSSQ, and PWSS). In addition, prior evidence shows that weight stigma is associated with psychological distress [62]. Therefore, we further hypothesized that the Chinese version of TAPAS would be associated with psychological distress measures (i.e., DASS-21).

For known-group validity, independent t-tests and Cohen’s d were used to examine if the TAPAS total score can distinguish between genders (i.e., men and women) and weight status groups (i.e., overweight and non-overweight). We hypothesized that women would have a higher TAPAS score than men based on the findings from the original TAPAS [12], and the overweight group would have a higher TAPAS score than the non-overweight group because the findings from the original TAPAS showed that BMI is associated with TAPAS [12].

Evidence suggests that weight status is associated with the TAPAS score, and the TAPAS score could be associated with psychological distress; therefore, a mediation model was tested to examine if the TAPAS score mediates the association between weight status and psychological distress. In the mediation model, Model 4 of Hayes’ Process Macro was used with the following set-ups: weight status as the independent variable, TAPAS score as the mediator, and DASS-21 as the dependent variable. 5000 bootstrapping resamples were used to examine the mediated effect of TAPAS: when the 95% bootstrapping confidence interval (CI) does not include 0, the mediated effect is supported.

## Results

### Participants’ demographics

Table 1 presents the participants’ characteristics. Among the 608 participants (mean [SD] age 29.10 [6.36] years), over half were women (*n* = 333; 54.8%), and slightly less than half were men (*n* = 273; 44.9%). Two participants self-reported their gender as other (0.3%). On average, the present sample had a height of 166.43 (SD = 8.50) cm, a weight of 66.51 (SD = 14.96) kg, a BMI of 23.89 (SD = 4.42) kg/m^2^, and 2.08 (SD = 1.95) days engaging in physical activity where breathing is somewhat harder than normal. Accordingly, nearly half of the present sample was classified as overweight (*n* = 268; 44.1%). Table 1 additionally reported the mean total scores of TAPAS, WBIS, WSSQ, PWSS, and DASS-21 for the present sample.

**Table 1:** Participant characteristics (*N* = 608)

	Mean (SD)	*n* (%)
Age (year)	29.10 (6.36)	/
Gender (Man)	/	273 (44.9)
Gender (Woman)	/	333 (54.8)
Gender (Others)	/	2 (0.3)
Height (cm)	166.43 (8.50)	/
Weight (kg)	66.51 (14.96)	/
Body mass index (kg/m^2^)	23.89 (4.42)	/
Overweight (24 kg/m^2^ or above)	/	268 (44.1)
Non-overweight (below 24 kg/m^2^)	/	340 (55.9)
Days of physical activity engagement per week^a^	2.08 (1.95)	/
0	/	151 (24.9)
1	/	124 (20.5)
2	/	133 (21.9)
3	/	82 (13.5)
4	/	35 (5.8)
5	/	36 (5.9)
6	/	11 (1.8)
7	/	34 (5.6)
TAPAS total score	25.36 (9.54)	/
WBIS total score	29.53 (7.05)	/
WSSQ total score	30.02 (9.73)	/
PWSS total score	1.34 (2.24)	/
DASS-21 total score	13.04 (10.95)	/

TAPAS = Tendency to Avoid Physical Activity and Sport Scale; WBIS = Weight Bias Internalization Scale (Cronbach’s α = 0.818; McDonald’s ω = 0.872); WSSQ = Weight Self-Stigma Questionnaire (Cronbach’s α = 0.922; McDonald’s ω = 0.923); PWSS = Perceived Weight Stigma Scale (Cronbach’s α = 0.859; McDonald’s ω = 0.867); DASS-21 = 21-item Depression, Anxiety, Stress Scale (Cronbach’s α = 0.936; McDonald’s ω = 0.937)

^a^ Physical activity engagement indicates that engaging physical activity that requires breathing being somewhat harder than normal

### Score distribution and internal consistency results

Table 2 presents the score distributions for the TAPAS items: the distributions could be considered normally distributed: skewness ranged between − 0.548 and 0.609; kurtosis ranged between − 1.126 and − 0.487. Moreover, the mean scores of the 10 TAPAS items ranged between 2.29 and 3.45. Cronbach’s α (0.95) and McDonald’s ω (0.95) of the TAPAS indicated excellent internal consistency, and the corrected item-to-total correlations of the TAPAS items were relatively strong (0.511 to 0.881).

**Table 2:** Score distributions of the Tendency to Avoid Physical Activity and Sport (TAPAS) scale

Item			*n* (%)			Mean (SD)	Skewness	Kurtosis	Factor loading	Item-total correlation
	Score 1	Score 2	Score 3	Score 4	Score 5					
TAPAS 1	169 (27.8)	213 (35.0)	117 (19.2)	101 (16.6)	8 (1.3)	2.29 (1.08)	0.450	-0.840	0.734	0.710
TAPAS 2	155 (25.5)	205 (33.7)	96 (15.8)	133 (21.9)	19 (3.1)	2.43 (1.18)	0.381	-1.017	0.787	0.765
TAPAS 3	178 (29.3)	201 (33.1)	104 (17.1)	98 (16.1)	27 (4.4)	2.33 (1.18)	0.565	-0.715	0.854	0.830
TAPAS 4	125 (20.6)	160 (26.3)	129 (21.2)	157 (25.8)	37 (6.1)	2.71 (1.23)	0.103	-1.126	0.801	0.785
TAPAS 5	100 (16.4)	147 (24.2)	141 (23.2)	177 (29.1)	43 (7.1)	2.86 (1.21)	-0.054	-1.072	0.726	0.712
TAPAS 6	188 (30.9)	210 (34.5)	110 (18.1)	85 (14.0)	15 (2.5)	2.23 (1.11)	0.609	-0.570	0.868	0.837
TAPAS 7	168 (27.6)	198 (32.6)	113 (18.6)	112 (18.4)	17 (2.8)	2.36 (1.15)	0.435	-0.892	0.904	0.873
TAPAS 8	164 (27.0)	219 (36.0)	102 (16.8)	102 (1.68)	21 (3.5)	2.34 (1.14)	0.547	-0.697	0.912	0.881
TAPAS 9	163 (26.8)	212 (34.9)	102 (16.8)	113 (18.6)	18 (3.0)	2.36 (2.15)	0.476	-0.841	0.903	0.871
TAPAS 10	56 (9.2)	65 (10.7)	15.6 (25.7)	211 (34.7)	120 (19.7)	3.45 (1.19)	-0.548	-0.487	0.519	0.511

Score 1 = strongly disagree; Score 2 = disagree; Score 3 = neutral; Score 4 = agree; Score 5 = strongly agree

Factor loadings were derived from one-factor structure confirmatory factor analysis using all participants’ data

### Confirmatory factor analysis with measurement invariance results

Except for the significant χ^2^ (χ^2^ = 57.362; df = 35; *p* = 0.01), the CFA results further supported the one-factor structure for the TAPAS: CFI = 0.998; TLI = 0.997; RMSEA = 0.032 (90% CI = 0.016, 0.047); SRMR = 0.042. Additionally, factor loadings of the TAPAS items were relatively strong (0.519 to 0.912) (Table 2). The one-factor structure of the TAPAS was further found to be invariant across two gender groups (i.e., men and women; those genders reported as other were not used for the invariance testing because of a too-small sample size) and across two weight status groups (i.e., overweight and non-overweight). All nested models showed satisfactory fit indices (CFI = 0.999 to 1.000; TLI = 1.000 to 1.001; RMSEA = 0.000 to 0.031; SRMR = 0.044 to 0.055).

Moreover, ΔCFI, ΔTLI, and ΔRMSEA were all 0.000 when comparing the metric invariance model with the configurable model and the scalar invariance model with the metric invariance model across gender groups. ΔSRMR was 0.002 when comparing the metric invariance model with the configurable model and was − 0.001 when comparing the scalar invariance model with the metric invariance model across gender groups. ΔCFI and ΔTLI were both − 0.001 when comparing the metric invariance model with the configurable model and the scalar invariance model with the metric invariance model across weight status groups. ΔRMSEA was 0.023 when comparing metric invariance model with configural model and was 0.008 when comparing scalar invariance model with metric invariance model across gender groups. ΔSRMR was 0.008 when comparing metric invariance model with configural model and was 0.001 when comparing scalar invariance model with metric invariance model across gender groups (Table 3).

**Table 3:** Multigroup confirmatory factor analysis of the Tendency to Avoid Physical Activity and Sport (TAPAS) scale

	Sex						Weight status				
	Man	Woman	M1	M2-M1	M3-M2		OW	nonOW	M1	M2-M1	M3-M2
χ^2^ or (Δχ^2^)	27.926	35.683	63.609	(6.022)	(6.748)		28.688	36.426	66.027	(25.946)	(20.881)
df or (Δdf)	35	35	70	(9)	(9)		35	35	70	(9)	(9)
p-value or (p-value for model comparisons)	0.80	0.44	0.69	(0.74)	(0.66)		0.77	0.40	0.61	(0.002)	(0.013)
CFI or (ΔCFI)	1.000	1.000	1.000	(0.000)	(0.000)		1.000	1.000	1.000	(-0.001)	(-0.001)
TLI or (ΔTLI)	1.002	1.000	1.001	(0.000)	(0.000)		1.001	1.000	1.000	(-0.001)	(-0.001)
RMSEA or (ΔRMSEA)	0.000	0.008	0.000	(0.000)	(0.000)		0.000	0.011	0.000	(0.023)	(0.008)
90% CI of RMSEA	0.000,0.029	0.000,0.040	0.000,0.027	/	/		0.000,0.032	0.000,0.041	0.000,0.029	/	/
SRMR or (ΔSRMR)	0.045	0.044	0.044	(0.002)	(-0.001)		0.044	0.047	0.046	(0.008)	(0.001)

*Abbreviation* M1 = configural model; M2 = metric invariance model; M3 = scalar invariance model; OW = overweight; nonOW = non-overweight. CFI = comparative fit index; TLI = Tucker-Lewis index; RMSEA = root mean square residual of approximation; SRMR = standardized root mean square residual; CI = confidence interval

### Concurrent validity, known-group, and mediation results

Concurrent validity of the TAPAS was evidenced by significant correlations with other external measures: *r* = 0.68 (*p* < 0.001) with WBIS; 0.66 (*p* < 0.001) with WSSQ; 0.33 (*p* < 0.001) with PWSS; and 0.37 (*p* < 0.001) with DASS-21 (Table 4).

**Table 4:** Concurrent validity of the Tendency to Avoid Physical Activity and Sport (TAPAS) scale (*N* = 608)

	TAPAS	WBIS	WSSQ	PWSS	DASS-21
TAPAS	--				
WBIS	0.68	--			
WSSQ	0.66	0.80	--		
PWSS	0.33	0.34	0.42	--	
DASS-21	0.37	0.38	0.37	0.33	--

*Note* All p-values < 0.001

*Abbreviation* WBIS = Weight Bias Internalization Scale; WSSQ = Weight Self-Stigma Questionnaire; PWSS = Perceived Weight Stigma Scale; DASS-21 = 21-item Depression, Anxiety, Stress Scale

Moreover, the known-group validity of the TAPAS was found in gender (men vs. women) and weight status (overweight vs. non-overweight) groups. Specifically, women reported significantly higher scores than men (Cohen’s *d* = 0.24; *p* = 0.004). People with overweight also reported significantly higher scores than those without overweight (Cohen’s *d* = 0.48; *p* < 0.001) (Table 5).

**Table 5:** Known-group validity of the Tendency to Avoid Physical Activity and Sport (TAPAS) scale (*N* = 608)

	*n*	Mean (SD)	t (*p*-value)	Cohen’s *d*
Gender	/	/	2.92 (0.004)	0.24
Man	273	24.13 (9.02)	/	/
Woman	333	26.39 (9.86)	/	/
Weight status	/	/	5.90 (< 0.001)	0.48
Overweight	268	27.87 (9.60)	/	/
Non-overweight	340	23.39 (9.04)	/	/

Lastly, the mediation model showed that TAPAS was a significant mediator in the association between weight status and psychological distress (unstandardized coefficient = 2.02; bootstrapping standard error = 0.40; 95% bootstrapping CI = 1.27, 2.83).

## Discussion

The present paper sought to develop a Traditional Chinese version of the TAPAS measure and establish its psychometric properties in an Asian population. All hypotheses were supported. The one-factor structure (Hypothesis 1) and internal consistency (Hypothesis 2) of the Chinese version of TAPAS were supported. The measurement invariance of the Chinese version of TAPAS was verified (Hypothesis 3) across genders (women vs. men) and weight status groups (overweight vs. non-overweight). The concurrent validity of TAPAS (Hypothesis 4) was supported via significant correlations with related construct criterion instruments. Moreover, the known-group validity of TAPAS (Hypothesis 5) was supported by significant TAPAS total score differences between men and women and between individuals who were overweight and those not overweight.

Similar to the findings from Bevan et al. [12], the present study found that the 10-item TAPAS in its Chinese version has a one-factor structure with good internal consistency. Moreover, the factor structure finding in the present study extends the exploratory factor analysis finding from Bevan et al. [12] to CFA. The one-factor structure also agrees with prior evidence using Rasch analysis on the TAPAS [63, 64]. The one-factor structure was further evidenced to be invariant across gender subgroups and weight status subgroups in the present study. Bevan et al. [12] found that the TAPAS was associated with weight stigma, and this association was replicated in the present findings (*r* = 0.68 with WBIS, 0.66 with WSSQ, and 0.33 with PWSS). We extended the concurrent validity from weight stigma measures to psychological distress measures (i.e., *r* = 0.37 with DASS-21 in the present findings). Consistent with the known-group validity findings from Bevan et al. [12], the present study found that women had significantly higher scores of TAPAS than men (Cohen’s d = 0.24). The present study further extended the known-group findings that overweight individuals had significantly higher TAPAS scores than those without (Cohen’s d = 0.48).

Supporting previous literature stating that weight status is associated with TAPAS score and TAPAS score could be associated with psychological distress; a mediation model was tested to examine if the TAPAS score mediated the association between weight status and psychological distress. Applying the Hayes’ Process Macro (Model 4), the mediator role of TAPAS in the association between weight status and psychological distress was shown in the present study. Hopefully, the addition of the mediation analysis solidifies the study and develops a strong base for further exploration. Because the TAPAS was found to be valid in the present Taiwanese sample, we compared the TAPAS scores of the present sample with Bevan et al.’s findings [12]. Bevan et al. [12] found that their Australian sample had a mean score of 19.63 (SD = 10.18), which is much lower than the present sample (mean [SD] score = 25.36 [9.54]). Moreover, a recent paper testing TAPAS psychometric properties on a mainland Chinese sample showed a mean TAPAS score of 23.44 (8.70) [65], indicating similar TAPAS scores between the two Chinese populations (i.e., Taiwanese and mainland Chinese). In other words, Western people (e.g., the Australian sample from Bevan et al.’s study [12]) and Eastern people (e.g., the present Taiwanese sample and the mainland Chinese sample from Saffari et al.’s study [61]) may have different levels of physical activity avoidance. However, more evidence is needed to understand better how culture may impact physical activity avoidance.

The TAPAS provides healthcare professionals and government policy-makers with a new tool for understanding the reasons for non-participation in physical activity and sports across different population groups. It also offers insights into the psychosocial factors that might be addressed to promote physical activity. Previous studies have mostly focused on structural and environmental factors associated with physical activity engagement [7–9]. However, some individuals, especially those who are overweight, might have psychosocial reasons for avoiding physical activity and sports [11, 12]. Therefore, the TAPAS can provide psychosocial factors to evaluate an individual’s tendency to avoid physical activity and sports. Accordingly, healthcare providers or government authorities can design appropriate programs to reduce psychosocial barriers (e.g., weight stigma or appearance concerns) for individuals to increase physical activity and use the TAPAS to evaluate the program’s effectiveness.

The present study has some limitations. First, the present study recruited Taiwanese youths aged between 20 and 40. Therefore, the generalizability of the present study’s findings might not be applied to other age groups (e.g., older people and adolescents). Second, the present study adopted a snowballing sampling method, which decreased the representativeness of the present sample. Indeed, the demographic information was not comparable to that of university students in Taiwan. Third, we did not examine the test-retest reliability of the Chinese version of the TAPAS. Therefore, whether the Chinese version of TAPAS possesses good reproducibility is unclear. Fourth, the responsiveness of the Chinese version of TAPAS was not examined. Therefore, future studies are needed to investigate whether the Chinese version of TAPAS can detect meaningful changes in the tendency of physical activity and sports avoidance. Fifth, given that this study applied a cross-sectional design, an important psychometric property of longitudinal invariance could not be examined. Future studies are needed to examine if the TAPAS could be interpreted invariantly across time. Sixth, the present study did not test content validity after the TAPAS was translated into Chinese. Seventh, the measures assessed in the present study were self-reported. Therefore, the present study’s findings, especially the concurrent validity findings, are subject to the common method variance bias. Finally, the present study did not have information regarding the participants’ daily exercise and sports engagement. Although we found that people who were overweight had higher TAPAS scores, this it could not be interpreted that they would have completely avoided physical activity and sports in their daily lives. Therefore, future studies are needed to identify the association between the tendency to avoid physical activity and sport with actual participation in physical activity and sport.

## Conclusion

The Chinese version of the TAPAS was found to be a valid and reliable instrument assessing individuals’ avoidance of physical activity and sport, paving the way for future cross-cultural comparisons. With the utilization of TAPAS, healthcare providers and government authorities in Taiwan can design programs to help Taiwanese youths reduce the impact of weight stigma and body image concerns on physical activity engagement. Specifically, the TAPAS can be used as an outcome evaluation to examine if intervention programs are effective. Apart from evaluating program effectiveness, the TAPAS can be used to assess how psychosocial factors may play a role in avoiding physical activity and sport.

## Data Availability

The datasets generated and/or analyzed during the current study are not publicly available due to ethical restrictions but are available from the corresponding author, C-Y.L, upon reasonable request.
