# Correction: Chinese version of the tendency to avoid physical activity and Sport (TAPAS) scale: testing unidimensionality, measurement invariance, concurrent validity, and known-group validity among Taiwanese youths

**DOI:** 10.1186/s40359-024-01912-5

**Published:** 2024-07-31

**Authors:** Yi-Ching Lin, Jung-Sheng Chen, Nadia Bevan, Kerry S. O’Brien, Carol Strong, Meng-Che Tsai, Xavier C. C. Fung, Ji-Kang Chen, I-Ching Lin, Janet D. Latner, Chung-Ying Lin

**Affiliations:** 1 Department of Early Childhood and Family Education, National Taipei University of Education, Taipei, Taiwan; 2 Department of Medical Research, E-Da Hospital, I-Shou University, Kaohsiung, Taiwan; 3 School of Social Sciences, Monash University, Melbourne, 3800 Australia; 4 Department of Public Health, College of Medicine, National Cheng Kung University, Tainan, Taiwan; 5 Department of Pediatrics, College of Medicine, National Cheng Kung University Hospital, National Cheng Kung University, Tainan, Taiwan; 6 Department of Medical Humanities and Social Medicine, College of Medicine, National Cheng Kung University, Tainan, Taiwan; 7 Department of Rehabilitation Sciences, Faculty of Health and Social Sciences, The Hong Kong Polytechnic University, Hung Hom, Hong Kong China; 8 Department of Social Work, Chinese University of Hong Kong, New Territories, Hong Kong China; 9 Department of Healthcare Administration, Department of Family Medicine, Asia University, Taichung, 41354 Taiwan; 10 Department of Family Medicine, Asia University Hospital, Taichung, 41354 Taiwan; 11 Department of Kinesiology, Health, and Leisure, Chienkuo Technology University, Changhua, 50094 Taiwan; 12 Department of Psychology, University of Hawaii, Honolulu, HI 96822 USA; 13 Institute of Allied Health Sciences, Departments of Occupational Therapy and Public Health, Biostatistics Consulting Center, College of Medicine, National Cheng Kung University Hospital, National Cheng Kung University, 1 University Rd, Tainan, 701401 Taiwan; 14 University of Religions and Denominations, Qom, Iran; 15 Biostatistics Consulting Center, College of Medicine, National Cheng Kung University Hospital, National Cheng Kung University, Tainan, Taiwan; 16 Faculty of Nursing, Universitas Padjadjaran, Sumedang, Indonesia

**Correction: BMC Psychology (2024) 12:381**

10.1186/s40359-024-01870-y

Following publication of the erratum, it came to the attention of the authors that affiliation 2 was incomplete: ‘I-Shou University’ had been omitted from the affiliation. The article has since been updated to fix this error. The authors thank you for reading this erratum and apologize for any inconvenience caused.

